# Nurses’ job stress and its impact on quality of life and caring behaviors: a cross-sectional study

**DOI:** 10.1186/s12912-022-00852-y

**Published:** 2022-03-31

**Authors:** Ali-Reza Babapour, Nasrin Gahassab-Mozaffari, Azita Fathnezhad-Kazemi

**Affiliations:** 1 Student Research Committee, Tabriz Medical sciences, Islamic Azad University, Tabriz, Iran; 2 Department of Nursing, Faculty of Nursing and Midwifery, Tabriz Medical Sciences, Islamic Azad University, Tabriz, Iran; 3 Department of Midwifery, Faculty of Nursing and Midwifery, Tabriz Medical Sciences, Islamic Azad University, Tabriz, Iran; 4 Women’s Reproductive and mental Health Research center, Tabriz Medical Sciences, Islamic Azad University, Tabriz, Iran

**Keywords:** Occupational stress, Nurse, Quality of life, Caring, Behavior

## Abstract

**Background:**

Nursing is considered a hard job and their work stresses can have negative effects on health and quality of life. The aim of this study was to investigate the correlation between job stress with quality of life and care behaviors in nurses.

**Methods:**

This cross-sectional survey design study was performed with the participation of 115 nurses working in two hospitals. The nurses were selected via the availability sampling method and data were collected by demographic characteristics, nurses ‘job stress, quality of life (SF12), and Caring Dimension Inventory questionnaires.

**Results:**

The mean (SD) total scores of job stress, quality of life and caring behavior were 2.77 (0.54), 56.64 (18.05) and 38.23 (9.39), respectively. There was a statistically significant and negative relationship between total job stress scores with quality of life (*r* = -0.44, *P* < 0.001, Medium effect) and caring behaviors (*r*=-0.26, *P* < 0.001, Small effect). Univariate linear regression showed that job stress alone could predict 27.9% of the changes in the total quality of life score (β =-0.534, SE = 0.051, R^2adj^ = 0.279, *P* < 0.001) and 4.9% of the changes in the total score of caring behaviors (β =-0.098, SE = 0.037, R^2adj^ = 0.049 *P* < 0.001).

**Conclusions:**

Job stress has a negative effect on the quality of life related to nurses’ health. It can also overshadow the performance of care and reduce such behaviors in nurses, which may be one of the factors affecting the outcome of patients.

## Background

Job stress is an interactive situation between the job situation and the working person in that job, which leads to changes in the individual’s psychological and physiological status and affects his/her normal performance [1]. Work-related stress can damage a person’s physical and mental health and ultimately have a negative effect on job productivity by increasing stress levels [2, 3]. Today, job stress has become a common and costly problem in the workplace and, according to the World Health Organization, a pervasive issue [4, 5].

Stress is determined as a major cause of 80% of all occupational injuries and 40% of the financial burden in the workplace according to the American Institute of Stress [4]. Nursing is known as a stressful job since it is associated with complex job demands and needs, and high expectations, excessive responsibility, and minimal authority have been identified as the main stressors [6]. The results of studies conducted in Iran show that 7.4% of nurses are absent each week due to mental fatigue or physical disability caused by work, which is 80% higher than other professional groups [7].

According to the statistics provided by the International Council of Nurses, the costs of work-related stress are estimated at $ 200–300 million annually in the United States, and nearly 90% of employees’ medical problems are attributed to job stress [8]. Job stress among nurses may affect their quality of life, and concurrently, the quality of care. The quality of life of nurses, who deal with human lives, is of particular importance since they can provide more effective services when they have a better quality of life [9]. Nurses are in close contact with patients and such factors as employment location, variety of hospitalized cases, lack of manpower, forced overtime hours, and the attitude of the ward manager can impose tremendous stress on nurses [10]. Although stress is a recognized component of modern nursing that is useful in small amounts, in the long run, chronic diseases, such as hypertension, lead to cardiovascular disease, and therefore, affect their quality of life [11]. Moreover, job stress causes job quit, co-workers conflict, health disorders, job dissatisfaction, reduced creativity, decreased professional satisfaction, reduced correct and timely decision-making, inadequacy and depression feelings, disgust and fatigue from work, reduced energy and work efficiency, and reduced quality of nursing care [12] and these items increase the likelihood of work-related injuries [13], regarding which, the results of numerous studies have shown that job stress has a direct or indirect effect on the provision of medical services [14]. Consequently, due to the inevitability of some stressors in the nursing profession, it is necessary to prevent their psychological and behavioral effects to improve nurses’ quality of life and their care behaviors [15]. The low caring behaviors can be influenced by several factors including individual and organizational factors like abilities, skills, job design and leadership style, respectively. Work stress can affect caring behavior nurses because of nurse, s excessive activity or workload and more duty [16]. Job related stress has as a result loss of compassion for patients and increased incidences of practice errors and therefore is unfavorably associated to quality of care [17]. Numerous studies reported that it has a direct or indirect effect on the delivery of care and on patient results [18, 19]. For instance, conflict with colleagues has been found to predict lower caring practice [5]. And another study explained Job satisfaction as personal satisfaction and satisfaction with nurse management was significantly associated with caring behavior [20]. However, in study conducted in Indonesia, the results showed that there was no association between workload and job stress with caring behaviors [16]. Data from Sarafis et al., show that work-related stress impacts nurses’ health-related quality of life negatively, furthermore, it can affect patient outcomes and they have emphasized the need for performing further research in this domain [5]. Assessment of possible basics and effects of work stress among nurses has been done [21]. However, factors such as individual differences and working conditions can affect it so that significant conflicts in work-related stress between nurses may be due to workplaces, general and cultural conditions [14].

Since nurses, as members of the healthcare system, make every effort to improve the quality of care and patients’ quality of life, it is crucial to address the factors affecting their quality of life [22] It is also important to assess the dimensions of quality of life and job stress, identify psychosocial risk factors, and plan for preventive interventions to increase the efficiency and effectiveness of nurses’ activities. According to our hypotheses, job stress leads to the decline of nurse’s physical and mental health status, while it is negatively affecting nurses’ caring behaviors. Therefore, the present study was designed to achieve the following goals:

1- Assessing the level of job stress, as well as the quality of life, and care behaviors in nurses. 2- Evaluating the relationship of between job stress with quality of life and care behaviors in nurses.

## Method

### Study Design and participants

This cross-sectional study was carried out in two teaching hospitals, namely the “Artesh” and “29 Bahman” hospitals, Tabriz, Iran, which are in cooperation with a non-governmental university in Tabriz, within December-January 2020. The employed nurses with at least one year of work experience and having contact with patients were entered into the study. The exclusion criteria were unwillingness to partake and failure to complete the questionnaire.

### Sample size and sampling method

The maximum sample size was considered after controlling research aims, so the highest sample size was calculated based on the study performed by Sarafis et al. [5] related to job stress scale with Considering 95% confidence coefficient, 90% statistical power, an acceptable error of 0.06 around the mean (m = 2.22), and the highest standard deviation of (0.65), therefore the necessary sample size was determined to be 96 cases. The final sample size was estimated at 115 subjects after considering a drop-out rate of 20%.

$$n=\frac{{({Z}_{1}-\frac{\alpha }{2})}^{2} \times {s}^{2}}{{d}^{2}}$$

### Procedure

Initially, the necessary permissions were obtained to conduct the research, which was followed by selecting the samples in different shifts using the availability sampling method. Afterward, the researcher referred to the hospital, explained the objectives of the study to the nurses, controlled the inclusion criteria, and obtained the participants’ satisfaction. The researcher provided the questionnaires to the samples to complete as a self-report. The sampling process was continued until reaching the calculated sample size.

### Measures

#### 1. Demographic characteristics checklist

This instrument included information about age, gender, education, marital status, shift status, employment status, and work experience.

#### 2. Expanded nursing stress scale (ENSS)

This 57-item scale consists of 9 subscales, measuring Death and Dying Stressors (7 items), conflict with physicians (5 items), Inadequate Emotional Preparation Stressors (3 items), Problems with Peers (6 items), Problems with Supervision (7 items), workload (9 items), Uncertainty Concerning Treatment (9 items), patients and their families (8 items), and discrimination (3 items). The items are rated on a 6-point Likert scale of 1 = I have no stress at all, 2 = sometimes I am stressed, 3 = often I am stressed, 4 = I am very stressed, and 5 = this situation does not include my duties, if a person is not faced with such a situation, the number zero is marked. The total scores are estimated at a range of 0-228, with higher scores indicating higher job stress in that particular area. To obtain the mean score of each subscale, the total score of each subscale is divided by the number of items. The range of mean values for the total score and subscales are obtained at 0–4 and no specific cut-off point is determined [23].

#### 3. Quality of Life Questionnaire-12 (SF-12)

SF-12 was constructed as a shorter alternative of the SF-36 Health Survey. SF-12, which measures physical and mental health status was used for the quality of life assessment. SF-12 includes 12 questions: 2 concerning physical functioning, 2 regarding role limitations caused by physical health problems, 1 question about bodily pain, 1 with reference to general health perceptions, 1 on vitality, 1 in regard to social functioning, 2 in relevance to role limitations because of emotional problems and 2 questions referring to general mental health [18]. To convert this score to the range of zero to 100, the raw score difference formula obtained from the minimum possible raw score divided by the difference of the maximum possible score of the minimum possible score is used. For the first time, Ware et al. [24] investigated the reliability and validity of this questionnaire and reported respective Cronbach’s alphas of 0.89 and 0.76 for physical health and mental health dimensions. Montazeri et al. investigated the reliability and validity of this scale in Iran and the reliability of the 12 items of physical and psychological elements was reported as 0.73 and 0.72, respectively [25].

#### 4. Caring dimension inventory (CDI-25)

The CDI consists of 25 core questions designed to gather perceptions of caring by asking subjects to indicate their agreement to statements about their nursing practice as constituting caring. The respondent is required to indicate on a 5-point Likert scale ranging from “strongly agree” to “strongly disagree” whether or not they perceive caring in this manner. Studies have shown that the CDI-25 is an instrument with acceptable psychometric properties. The tool includes five dimensions: psychosocial (10 items), physical-technical (11 items), professional (1 item), unnecessary (1 item) and inappropriate (2 items). Items 3 and 16 are scored in reverse, so that the strongly agree and strongly disagree options are given the lowest and highest scores, respectively [26].

The reliability of this instrument was determined using two methods of calculating internal consistency by Cronbach’s alpha coefficient and intraclass correlation coefficient (ICC) by test-retest on 20 nurses. The Cronbach’s alpha coefficient and ICC (confidence interval of 95%) were calculated for job stress quionnaires at 0.78 and 0.82 (0.76–0.84), for quality of life at 0.87 and 0.89 (0.82–0.92), and caring dimension 0.81 and 0.85 (0.79–0.87), respectively.

### Data analysis

SPSS-22 software is used to analyze the quantitative data. Sociodemographic, ENSS, SF-12, CDI-25 questionnaires score described by frequency (percent), as well as mean (Standard Deviation). The association between Sociodemographic with ENSS, SF-12 and CDI-25 determined using the t-test, ANOVA and their nonparametric equivalents for abnormally distributed variables (Mann-Whitne U test, Kruskal-Wallis H test). The associations of two continuous variables were analyzed by Spearman correlation tests in the bivariate analysis. Then, independent variables, with *P* ≤ 0.05 on bivariate tests inserted into the multivariate linear regression model (enter method). The normality of quantitative data was measured based on kurtosis and skewness, Since the SF-12 and CBI scores were not normally distributed, this values were first converted by use of a natural logarithm(Ln) transformation which yielded distributions that did not significantly deviate from normality then It was used in linear regression. All tests were 2-sided.

## Results

The statistical population of the study consisted of 115 nurses (100% response rate) with mean age and work experience scores of 31.81(8.18) and 7.95(7.35), respectively. The demographic information of the samples is presented in Table 1. The majority of participants (61.7%) were female, more than half of the subjects were married, and more than three-quarters of them had a bachelor’s degree. The employment status of most of the participants (63.5%) was permanent and most of them worked rotating shifts. The participants were selected from a range of different departments and 39.1% were working Internal medicine department.

**Table 1:** Sample characteristics (*n* = 115)

variable	(%) n	Job stress		SF-12^a^		Care behavior	
		**Mean(SD** ^**a**^ **)**	***P*-value**	**Mean(SD)**	***P*-value**	**Mean(SD)**	***P*-value**
**Gender**							
Male	44(38.3)	2.49 (0.50)	< 0.001	62.18(16.08)	0.01^b^	41.64(11.24)	0.007
Women	71 (61.7)	2.95(0.49)		53.21(18.44)		36.11(7.36)	
**Age (year)**							
< 25	26 (22.6)	2.61 (0.39)	< 0.001	58.55(14.84)	0.879^c^	42.58(10.58)	0.090
25–29	37 (32.2)	2.67(0.52)		58.41(19.01)		37.30(10.58)	
30–34	20 (17.4)	2.89 (0.66)		57.36(21.65)		37.10(7.29)	
35–39	12 (10.4)	3.07 (0.48)		54.63(15.13)		39.50(14.41)	
40–44	5 (4.3)	3.10(0.08)		49.44(18.25)		37.60(7.12)	
45–49	9 (7.8)	2.43 (0.44)		52.78(14.76)		32.44(5.15)	
> 50	6 (5.2)	3.41 (0.49)		50.93(25.49)		35.50(9.52)	
**Marital status**							
single	53 (46.1)	2.69(0.47)	0.149	57.23(18.18)	0.824	40.30(9.91)	0.012
married	62 (53.9)	2.84(0.60)		56.14(18.06)		36.45(8.61)	
**Education status**							
Bachelor	107 (93.0)	2.76(0.56)	0.353	56.26(18.35)	0.404	38.28(9.61)	0.822
Masters	8 (7.0)	2.95(0.25)		61.81(13.00)		37.50(5.97)	
**Employment Status**							
Official	73 (63.5)	2.86(0.55)	0.031	55.56(17.83)	0.397	37.88(9.49)	0.601
Contractual	42 (36.5)	2.63(0.52)		58.53(18.48)		38.83(9.31)	
**Working position**							
Supervisor	6 (5.2)	2.61 (0.60)	0.575	2.61 (0.60)	0.404	33.50(5.08)	0.339
head-nurse	8 (7.0)	2.92 (0.27)		2.92 (0.27)		36.65 (8.10)	
Nurse	101 (87.8)	2.77 (0.56)		2.77 (0.56)		38.65 (9.64)	
**Shift**							
Morning	14 (12.2)	2.79(0.36)	0.966	62.50(11.15)	0.980	33.71(6.79)	0.735
Rotated	101 (87.8)	2.77(0.57)		55.83(18.70)		38.85(9.55)	
**The number of night shifts per month**							
0	8 (7.0)	2.88(0.24)	0.477	52.43(19.83)	0.653	52.43(19.83)	0.791
4 ≥	7 (6.0)	2.98(0.16)		61.11(11.45)		57.25(18.05)	
> 4	100 (87.0)	2.75(0.57)		56.67(18.34)		56.88(18.05)	
**Nurse’s department**							
Male Internal medicine	23 (20.0)	22.94 (5.33)	0.002	50.12(15.20)	0.071	6.91(9.41)	0.300
Female Internal medicine	22 (19.1)	23.74 (4.30)		58.46(22.32)		37.45(8.16)	
ICU ^b^	11 (9.6)	28.70 (3.35)		48.99(19.49)		38.45(5.57)	
CCU ^c^	6 (5.2)	27.53 (0.40)		69.44(0.00)		34.33(9.18)	
Diyaliz	11 (9.6)	22.78 (5.54)		68.18(10.56)		44.55(14.61)	
Surgery	23 (20.0)	25.20 (5.30)		57.13(20.35)		39.22(10.06)	
Emergency	13 (11.3)	28.31 (3.02)		55.13(15.70)		38.23(9.39)	
Nurse director office	6 (5.2)	23.68 (5.58)		56.48(9.07)		33.50(5.08)	

^a^ Standard Deviation, ^b^ Intensive care units, ^c^ Coronary care unit

### Mean values of the ENSS, SF-12 and CDI-25

The data related to the main study variables are tabulated in Table 2. The mean(SD) total scores of job stress, quality of life, and caring behavior were obtained at 2.77(0.54), 56.64(18.05), and 38.23(9.39), respectively. Among the job stress subscales, the highest scores were related to death and dying stressors and inadequate emotional preparation. In this same vein, the lowest score was related to the discrimination subscale. Regarding the quality of life subscale, the highest score was obtained in the field of physical health (m = 62.97 and SD = 19.42). The evaluation of the relationship between demographic characteristics and study parameters showed that job stress had a significant relationship with age, gender, employment status, and nurse’s department. In this respect, it was found that the mean scores of job stress were higher in women than in men (2.95 vs. 2.49) and in permanent nurses than in casual ones (2.86 vs. 2.63). Also, the participants who work in ICU and emergency departments have a high level of stress compared to the others. Among the demographic characteristics, only gender had a significant relationship with the quality of life, and the total mean score of quality of life was higher in men than in women (62.18 vs. 53.21). The relationship between demographic characteristics and caring behaviors showed that the total score of caring behaviors was significantly higher in men than in women (41.64 vs. 36.11) and in single cases than in married ones (40.30 vs. 36.45) (Table 1).

**Table 2:** Mean values of study parameters

Variable	Mean(SD)
**ENSS**	
Death and Dying Stressors	3.04 (0.60)
Inadequate Emotional Preparation Stressors	3.08 (0.90)
Problems with Supervision Stressors	3.07 (0.83)
Conflict with Physician Stressors	2.87 (0.74)
Patient and Family Stressors	2.68 (0.56)
Uncertainty Concerning Treatment Stressors	2.66 (0.59)
Workload Stressors	2.63 (0.57)
Problems with Peers Stressors	2.51 (0.66)
Discrimination Stressors	2.23 (0.88)
Total stress score	2.77 (0.54)
**SF-12**	
Physical component score	62.97 (19.42)
Mental component score	52.51 (19.92)
Total SF-12 score	56.64 (18.05)
**CBI**	
Physical-technical	16.39 (4.26)
Psycho-social dimension	14.74 (4.35)
Inappropriate	5.23 (1.02)
unnecessary	1.39 (0.60)
Professional	1.22 (0.45)
Total CBI score	38.23 (9.39)

### Correlation between ENSS with SF-12 and CDI-25

The data related to the relationship between the main variables of the study are summarized in Table 3. It was revealed that the total score of quality of life was negatively correlated with all components of job stress, which was statistically significant and moderate. Furthermore, the psychological domain of quality of life had a negative correlation with all components of job stress. It was also found that there was a significant and negative relationship between the physical domain and most dimensions of job stress, except for problems with peers and death and dying stressors (Table 3).

**Table 3:** Coloration between Job Stress with Quality of life and care behaviors

Job stress	Quality of life			caring behaviors					
	**Physical component**	**Mental component**	**Total SF-12**	**Unnecessary**	**Physical-technical**	**Professional**	**Inappropriate**	**psychosocial**	**Total score**
**Total stress score**	0.21-^*^	0.55-^**^	-0.44^**^	0.22-^*^	0.09-	0.23-^*^	0.06-	0.30-^**^	0.26-^**^
Conflict with Physician Stressors	0.20-^*^	0.50-^**^	0.41-^**^	0.15-	0.03	0.13-	0.08	0.37-^**^	0.21-^*^
Inadequate Emotional Preparation Stressors	0.12-	0.47-^**^	0.36-^**^	0.13-	0.11	0.15-	0.12	0.23-^*^	0.03-
Problems with Peers Stressors	0.11-	0.48-^**^	0.35-^**^	0.1-	0.02	0.08-	0.04-	0.24-^**^	0.122-
Problems with Supervision Stressors	0.30-^*^	0.37-^**^	0.35-^**^	0.27-^*^	0.27-^*^	0.08-	0.11-	0.18-	0.33-^*^
Workload Stressors	0.21-^*^	0.38-^**^	0.33-^**^	0.12-	0.00	0.09-	0.05	0.29-^**^	0.17-
Uncertainty Concerning Treatment Stressors	0.18-	0.48-^**^	0.38-^**^	0.07-	0.03	0.03-	0.05	0.29- ^**^	0.14-
Patient and Family Stressors	0.27-^*^	0.50-^**^	0.43-^**^	0.16-	0.03-	0.15-	0.02-	0.42-^**^	0.23-^*^
Discrimination Stressors	0.19-^*^	0.22-^*^	0.22-^*^	0.15-	0.07-	0.19-^*^	0.10-	0.14-	0.17-
Death and Dying Stressors	-0.16	0.35-^**^	0.30-^*^	0.06-	0.01	0.27-^**^	0.05-	0.28-^*^	0.16-

^*^P < 0.05

^**^P < 0.001

Based on the results of the study, the total score of caring behaviors had a negative correlation with the total and components scores of job stress. However, this correlation was significant merely with the total score, conflict with physicians, Problems with Supervision, and patients and their families, which was weak. In addition, a significant and negative relationship was found between most areas of caring behaviors and the dimensions of job stress. Nonetheless, this correlation was not statistically significant in between most aspects of both scale, and there was a positive relationship between most dimensions of job stress and inappropriate behaviors (Table 3).

The results of univariate linear regression analysis showed that job stress alone could explain 27.9% of the changes in the total score of quality of life (R^2^_adj_ = 0.279, *P* < 0.001) and the negative relationship between these two variables indicated that an increase in the standard deviation of the total job stress score, could decrease the quality of life by 0.534 (β=-0.534, SE = 0.051, *P* < 0.001). Moreover, according to the multivariate linear regression, job stress (β=- 0.514, *P* < 0.001) and gender (β=-0.029, *P* = 0.745) were predictive factors for quality of life, and the model 2 explained 27.4% changes in quality of life (*P* < 0.001) (Table 4).

**Table 4:** Effect of ENSS and demographic characteristics on SF-12 based on univariate and multivariate linear regression

Predictors		R	R^2^	R^2^_adj_	B	S.E	β	95%CI	F	*P*-Value
**Model 1**	ENSS	0.534	0.286	0.279	-0.343	0.051	-0.534	-0.44 to -0.24	45.157	< 0.001
**Model 2**		0.527	0.277	0.264					21.484	< 0.001
	constant				105.33	7.726		90.03 to 120.64		< 0.001
	ENSS				-16.930	2.911	-0.514	-0.44 to -0.22		< 0.001
	Gender				-1.066	3.267	-0.029	-7.53 to 5.04		0.745

Based on the findings of Table 5, job stress was able to predict 4.9% of the changes in the total score of caring behaviors (R^2^_adj_ = 0.049) and there was a negative relationship between these two variables; in other words, a 1 standard deviation increase in the total score of job stress led to the 0.098 decrease of caring behaviors rate (β=-0.098, *P* < 0.001). The results of multivariate linear regression showed that independent variables could explain 9.2% of the dependent variable changes (*P* = 0.002), however job stress (β=-0.059, *P* = 0.146), gender (β=-0.084, *P* = 0.075), and marital status (β=-0.064, *P* = 0.127) weren’t significant effect of caring behaviors.

**Table 5:** Effect of ENSS and demographic characteristics on CBI based on univariate and multivariate linear regression

Predictors		R	R^2^	R^2^_adj_	B	S.E	β	95%CI	F	*P*-value
**Model 1**	ENSS	0.239	0.057	0.049	-0.098	0.037	-0.239	-0.17 to 0.02	6.837	< 0.001
**Model 2**		0.341	0.116	0.092					4.857	0.002
	constant				4.014	0.115		3.48 to 4.23		< 0.001
	ENSS				-0.059	0.040	-0.144	-0.13 to 0.021		0.146
	Gender				-0.084	0.046	-0.182	-0.17 to 0.00		0.075
	marital status				-0.064	0.042	-0.143	-0.14 to 0.01		0.127

## Discussion

This study aimed to explore the status of job stress and examine its relationship with nurses’ quality of life and caring behavior. The results of the study indicated a negative relationship among the main components of the research. In this regard, the mean total score of job stress was higher than normal, which was slightly higher than that found in a study conducted in Greece. However, the findings of most studies have reported high levels of anxiety and stress among nursing staff. It was revealed that job stress was higher in females than in males, which could be attributed to their different roles in daily life. Nevertheless, in some studies, no significant difference was found between gender and job stress [27, 28]. However, the lack of relationship between gender and stress levels in the mentioned studies may be due to a large number of female than men participants.

In the present study, no significant difference was found between marital status and the stress level, which was inconsistent with that of a study performed by Mehrabi et al. [29] the reason may be due to the fact that married individuals’ more involvement in life issues and the impact of other life matters that can affect job stress. In our study, an increase in stress level was also observed with aging, which may be due to the effect of job burnout in individuals. In the study of Abarghouei et al., there was no significant relationship between age and job stress, but there was a direct relationship between job stress and job history. Also, with increasing work experience, the rate of burnout was higher and there was relationship between job stress and job burnout [30]. Long-term job stress has been shown to lead to burnout which is often used by the health care personnel synonymously with occupational stress [31, 32]. However, no relationship was found between age and stress in several studies [28, 33, 34], which can be attributed to the fact that, in our study, 50% of the participants were under 30 years old. In addition, the level of job stress was higher in permanent employees than in contract ones, which can be attributed to a higher sense of responsibility and more duties. Also, in the present study, no significant difference was found among different shifts. Data from Arkerstedt et al. support the idea that nighttime work is hazardous to a person’s wellbeing [35] and different work shifts are considered one of the sources of stress among nurses [36], however, the results of studies on this subject are different. So that in some studies, stress levels were reported to be high in night shift nurses [37, 38] and in others, stress was reported to be high in morning shift nurses [36, 39]. overall, it should be acknowledged that the sources of job stress and its level of effect are different according to the working conditions, working department, and culture of each society so nurses may have different levels of job stress and influencing factors due to different working conditions and the level of support provided.

According to the findings of our study, most occupational stressors were related to death and Death and Dying Stressors and insufficient emotional preparedness. The first occupational stressors were found to be death and suffering, which was consistent with the results of studies conducted in Greece [40] and the Philippines [41], reporting that such stress is probably rooted in the inability to prevent death. The second most important factor of job stress in the present study was found to be insufficient emotional preparedness, which in a study performed by Sarafis [5], this second cause of job stress was revealed to be the conflict with the patient and family. However, in our study, this factor was reported as the fifth cause of job stress. Insufficient emotional preparedness might have been created due to the sudden outbreak of COVID-19 at the time of performing the present study.

The third source of job stress was related to the problems with the supervisor, which was also reported as the third stressor in the study carried out by Sarafis et al. [5]. Based on the findings of a study conducted in Japan, poor support from the supervisor was associated with depression [42]. English researchers have also reported that the lack of adequate support from nursing managers leads to a significant increase in stress, whereas support with supervision causes a reduction in job stress [43]. The least stressful factor was revealed to be discrimination, which was in agreement with the results of other studies. Park and Haq introduced the lack of receiving rewards and encouragement as the main cause of stress among nurses, which can be attributed to the differences in the statistical population and used questionnaires between these two studies [44, 45].

According to another finding of the present study, the total score of quality of life and its dimensions was obtained in the middle range, and the mean score of the psychological dimension was lower than the physical dimension. A review of the literature indicated that the mean values of the total score and dimensions of quality of life in our study were lower than those in other studies [7, 46, 47]. This discrepancy can be regarded as the time of performing the research, which coincided with the onset of coronavirus. Based on the findings of researchers, nurses are under various mental and physical pressures depending on their job status. The reason for such stress can be due to nurses’ workload during the day since they are responsible to take care of several patients simultaneously, the repetition of which on consecutive days causes physical and psychological damages, and ultimately, affects the quality of life [4]. There was a significant inverse relationship among all dimensions of job stress with total quality of life and psychological and physical dimensions, which was consistent with the results of other studies conducted in this domain [7, 48]. The results revealed that job stress had a moderate and weak relationship with psychological and physical dimensions, respectively. Also, 27.9% of the changes in the total score of quality of life was related to job stress and this relation was negatively significant (β=-0.534, *P* < 0.001). According to the previous report, job stress is associated with low self-esteem, depression, anxiety, and feelings of inadequacy, which is considered a major risk factor for mild psychiatric illness [49]. Moreover, it was revealed that heavy workload, long working hours, lack of support, and Inability to quit work and not having enough rest can cause physical harm to the nurses, reduce their quality of life, and increase stress and tension in the workplace [50]. Also, researchers shown that job stress was as an independent predictor of quality of life related to mental component (β=-4.98, *P* < 0.001), and stress resulting from conflicts with supervisors was independently associated with mental health [5]. which is consistent with our study. According to researchers, good mental health increases trust and cooperation and control stressors [51]. Mental well-being and capacity to cope with stressful situation is important [52]. The psychological well-being is related to the higher using of the different coping strategies [53] and cultivating an environment of trust may provide organizations with a strategy to improve levels of mental health and satisfaction among their employees [54].

According to the results, nurses have paid more attention to such caring behaviors as writing reports, wearing clean and tidy uniforms, monitoring vital signs, and reporting the patient’s condition to the superior nurse, which is a technical-professional. However, assisting patients in daily activities, sitting at the patient’s bedside and talking to him/her, maintaining the patient’s privacy, maintaining professional competence, and listening to the patient were considered the least important. Overall, 4.9% of the changes in the total score of caring behaviors was related to occupational stress and the relation between to variables was negative (β=-0.098, *P* < 0.001). The comparison of results of this study with those of similar studies performed in Spain and England indicated the existence of differences in the understanding of nurses’ caring behaviors [55]. However, this finding was consistent with that of studies conducted in Iran [56, 57]. Factors influencing caring behaviors can be rooted in nursing education [57]. Paying too much attention to physical care during education, increasing the workload in the ward, a large number of patients per nurse in each shift have a great impact on the performance of nurses at the patient’s bedside [58]. Job stress were significant and independent predictors of total caring behaviors and its subscales.

Researchers have attributed these discrepancies to the cultural differences of societies [49]. The data analysis showed a weak inverse relationship between job stress and different dimensions of caring behaviors. It should be noted that the increase in scores in various dimensions of job stress had a significant negative relationship with the psychosocial domain, which was moderate. Apparently, psychosocial support of the patient decreases with an increase in such stressors as conflict with physicians, patient and family, and increased workload.

According to researchers, job stress is a physical-psychological syndrome accompanied by fatigue that leads to negative behaviors and attitudes toward oneself, work, family, and patients, and causes ineffective activity and absenteeism, immorality, and job dissatisfaction, seemingly stemming from nurses’ mental stress and lack of concentration [51]. Excessive job stress has negative impacts on nurses’ psychological well-being and reduces their work productivity. The results of the present study have been confirmed by the reports of other researchers; in this respect, the job stress of healthcare workers has a relationship with their low job satisfaction, negative attitude towards own job, and negative consequences on the quality of caregiving [59, 60].

### Limitations

One of the limitations of this study was related to the type of this research since it is not possible to correctly determine causal relationships in cross-sectional studies. Furthermore, the use of the availability sampling method can be one of the limitations of the study; however, the performance of this study in two teaching hospitals contributed to the effective generalization of the results. Moreover, the non-significant result especially between groups may be due to the low sample size and because of a small sample size, the number of predictive variables to be included in the regression models was limited. Since the present study was conducted at the time of the outbreak of COVID-19, it can be a confounding factor on the main variables of the study, namely job stress, quality of life, and caring behaviors, the effects of which have not been investigated. For future study in this area, these limitations described above have important implications for similar projects.

## Conclusions

In general, the findings of this study showed that employed nurses had higher levels of perceived job stress that can have negative effects on their quality of life and caring behaviors. Job stress can endanger the physical and mental health of nurses, decrease energy and work efficiency, and fail to provide proper nursing care, which ultimately has a negative impact on patient outcomes. Therefore, it is required to investigate the stressors and effective planning to eliminate these factors. The provision of educational programs to the proper introduction of this profession to the community can increase awareness about the nurses’ problems and concerns, and ultimately, improve their quality of life. Nevertheless, it is recommended the initial management be performed at the organizational level. Purposeful education in university on nursing professional values is essential and hospital managers can improve nurses’ quality of life and caring behaviors by providing cognitive-behavioral intervention programs with the aim of identifying sources of stress in the workplace and providing soft skill programs such as team working, behavioral and communication skills and teaching effective coping strategies to reduce stressors.

## Data Availability

The datasets are available from the corresponding authors on request.

## Abbreviations

ENSS: Expanded Nursing Stress Scale.
SF-12: Quality of Life Questionnaire-12.
CDI-25: Caring Dimension Inventory

## References

[1] Richardson KM, Rothstein HR (2008). Effects of occupational stress management intervention programs: a meta-analysis. J Occup Health Psychol.

[2] Unaldi Baydin N, Tiryaki Sen H, Kartoglu Gurler S, Dalli B, Harmanci Seren AK (2020). A study on the relationship between nurses’ compulsory citizenship behaviours and job stress. J Nurs Manag.

[3] Labrague LJ, Nwafor CE, Tsaras K (2020). Influence of toxic and transformational leadership practices on nurses’ job satisfaction, job stress, absenteeism and turnover intention: A cross-sectional study. J Nurs Manag.

[4] Sveinsdottir H, Biering P, Ramel A (2006). Occupational stress, job satisfaction, and working environment among Icelandic nurses: a cross-sectional questionnaire survey. Int J Nurs Stud.

[5] Sarafis P, Rousaki E, Tsounis A, Malliarou M, Lahana L, Bamidis P, Niakas D, Papastavrou E (2016). The impact of occupational stress on nurses’ caring behaviors and their health related quality of life. BMC Nurs.

[6] Jacobs AC, Lourens M (2016). Emotional challenges faced by nurses when taking care of children in a private hospital in South Africa. Africa J Nurs Midwifery.

[7] Nasiry Zarrin Ghabaee N, Talebpour Amir F, Hosseini Velshkolaei M, Rajabzadeh R (2016). Quality of life and its relationship to the Job stress in among nursing staff in Hospitals of Sari, in 2015. J Nursing Educ.

[8] Hassard J, Teoh KR, Visockaite G, Dewe P, Cox T (2018). The cost of work-related stress to society: A systematic review. J Occup Health Psychol.

[9] Layali I, Ghajar M, Abedini E, Emadian SO, joulaei M (2019). Role of Job Stressors on Quality of Life in Nurses. J Mazandaran Univ Med Sci.

[10] Geiger-Brown J, Lipscomb J (2010). The health care work environment and adverse health and safety consequences for nurses. Annu Rev Nurs Res.

[11] Bahrami M (2016). Nurses’ quality of life in medical-surgical wards of an oncology center affiliated to the Isfahan University of Medical Sciences. Nurs J Vulnerable.

[12] Valiei S, Rezaei M, Rezaei K (2013). The relationship between personality characteristics and Nursing occupational stress. Iran J Psychiatr Nurs.

[13] Shareinia H, Khuniki F, Bloochi Beydokhti T (2018). Comparison between job stress among emergency department nurses with nurses of other departments. Q J Nurs Manag.

[14] Parveen R, Hussain M, Afzal M, Parveen MK, Majeed MI, Tahira F, Sabir M (2017). The impact of occupational stress on nurses caring behavior and their health related quality of life. Saudi J Med Pharm Sci.

[15] Abedini R, Choobineh A, Hasanzadeh J (2013). Musculoskeletal load assessment in hospital nurses with patient transfer activity. Int J Occu Hygiene.

[16] Rizkianti I, Haryani A (2020). The Relationship Between Workload and Work Stress With Caring Behavior Of Nurses in Inpatient Rooms. Jurnal Aisyah: Jurnal Ilmu Kesehatan.

[17] Aiken LH, Cimiotti JP, Sloane DM, Smith HL, Flynn L, Neff DF (2012). Effects of nurse staffing and nurse education on patient deaths in hospitals with different nurse work environments. J Nurs Admin.

[18] O’Connell E, Landers M (2008). The importance of critical care nurses’ caring behaviours as perceived by nurses and relatives. Intensive Crit Care Nurs.

[19] Esmaiel Hajinezhad M, Azodi P (2014). Nurse caring behaviors from patients’ and nurses’ perspective: A comparative study. Eur Online J Nat Soc Sci.

[20] Oluma A, Abadiga M (2020). Caring behavior and associated factors among nurses working in Jimma University specialized hospital, Oromia, Southwest Ethiopia, 2019. BMC Nurs.

[21] Brown H, Zijlstra F, Lyons E (2006). The psychological effects of organizational restructuring on nurses. J Adv Nurs.

[22] Hsu MY, Kernohan G (2006). Dimensions of hospital nurses’ quality of working life. J Adv Nurs.

[23] French SE, Lenton R, Walters V, Eyles J (2000). An empirical evaluation of an expanded nursing stress scale. J Nurs Measurement.

[24] Ware Jr JE, Kosinski M, Keller SD (1996). A 12-Item Short-Form Health Survey: construction of scales and preliminary tests of reliability and validity. Med Care.

[25] Montazeri A, Vahdaninia M, Mousavi SJ, Omidvari S (2009). The Iranian version of 12-item Short Form Health Survey (SF-12): factor structure, internal consistency and construct validity. BMC Public Health.

[26] Watson R, Deary IJ, Lea A (1999). A longitudinal study into the perceptions of caring among student nurses using multivariate analysis of the Caring Dimensions Inventory. J Adv Nurs.

[27] Delirrooyfard A, Masoumi K, Forouzan A (2015). Occupational stress among emergency department nurses of Golestan and Emam Khomeini hospitals in Ahvaz. Jundishapur Sci Med J.

[28] Mojdeh S, Sabet B, Irani MD, Hajian E, Malbousizadeh M: Relationship of nurse’s stress with environmental-occupational factors. Iran J Nurs Midwifery Res. 2008;13(1):1–5.

[29] Mehrabi T, Parvin N, Yazdani M, Rafat NA: A study of the severity of some occupational stresses in nurses. Iranian Journal of Nursing and Midwifery Research 2008, 12(1).

[30] Abarghouei MR, Sorbi MH, Abarghouei M, Bidaki R, Yazdanpoor S (2016). A study of job stress and burnout and related factors in the hospital personnel of Iran. Electron Phys.

[31] Estryn-Behar M, Doppia M, Guetarni K, Fry C, Machet G, Pelloux P, Aune I, Muster D, Lassaunière J, Prudhomme C (2011). Emergency physicians accumulate more stress factors than other physicians–results from the French SESMAT study. Emerg Med J.

[32] Tür FÇ, Toker İ, Şaşmaz CT, Hacar S, Türe B (2016). Occupational stress experienced by residents and faculty physicians on night shifts. Scand J Trauma Resusc Emerg Med.

[33] Valiei S, Rezaei M, Rezaei K, Ghanei Gheshlagh r (2013). The relationship between personality characteristics and Nursing occupational stress. J Nurs Educ.

[34] Mortaghi Ghasemi M, Ghahremani Z, Vahedian Azimi A (2011). Nurses Job Stress in Therapeutic Educational Centers in Zanjan. J Res Dev Nurs Midwife.

[35] Åkerstedt T (1990). Psychological and psychophysiological effects of shift work. Scand J Work Environ Health.

[36] Ameri M, Fadaee Aghdam N, Khajeh M, Goli S, Baha R (2021). Factors Related To Job Stress In Different Work Shifts Of Nurses Working In Hospitals Affiliated To Shahroud University Of Medical Sciences. Nurs Midwif J.

[37] Gheshlagh R, Parizad N, Dalvand S, Zarei M, Farajzadeh M, Karami M, Sayehmiri K (2017). The prevalence of job stress among nurses in Iran: A meta-analysis study. Nurs Midwif Stud.

[38] Mashak B, Farhand B, Moghadam S, Pazhoom Z, Hajalikhani T, Taghipoor N, Soltannezhad N, Shahnavaz N, Farid M (2015). Relationship Between Job Stress Among Nurses with Their General Health Status in Kamali Hospital in 1392. Alborz Univ Med J.

[39] Rocha MCPd, Martino MMFD (2010). Stress and sleep quality of nurses working different hospital shifts. Revista da Escola de Enfermagem da USP.

[40] Chatzigianni D, Tsounis A, Markopoulos N, Sarafis P (2018). Occupational stress experienced by nurses working in a Greek Regional Hospital: A cross-sectional study. Iran J Nurs Midwife Res.

[41] Andal EM (2006). Pilot Study Quantifying Filipino Nurses’ Perception of Stress. Californian J Health Promotion.

[42] Kawano Y (2008). Association of job-related stress factors with psychological and somatic symptoms among Japanese hospital nurses: Effect of departmental environment in acute care hospitals. J Occu Health.

[43] McGilton KS, Hall LM, Wodchis WP, Petroz U (2007). Supervisory support, job stress, and job satisfaction among long-term care nursing staff. JONA.

[44] Haq Z, Iqbal Z, Rahman A (2008). Job stress among community health workers: a multi-method study from Pakistan. Int J Mental Health Syst.

[45] Park Y-M, Kim SY (2013). Impacts of job stress and cognitive failure on patient safety incidents among hospital nurses. Safety Health Work.

[46] Chen YM, Chen SH, Tsai CY, Lo LY (2007). Role stress and job satisfaction for nurse specialists. J Adv Nurs.

[47] Jafari S, Batebi A, Hosseini M, Ebrahimpoor M, Shojaei F, Vaezi M (2012). The Effects of occupational stress on quality of life and associated factors among hospital nurses in Iran. J Soc Dev Sci.

[48] Kordi M, Mohamadirizi S, Shakeri MT, Modares Gharavi M, Salehi Fadardi J (2014). The relationship between occupational stress and work ability among midwives in Mashhad, Iran. J Midwife Reprod Health.

[49] Khaganizade M, Ebadi A, Siratinaier M, Rahmani M (2008). Assessmen of correlation job stress and occupational quality of life in nursing of military Hospitals. Mil Med J.

[50] McGrath A, Reid N, Boore J (2003). Occupational stress in nursing. Int J Nurs Stud.

[51] Roh H, Lee D, Kim Y (2014). Prevalence of work-related musculoskeletal symptoms and their associations with job stress in female caregivers living in South Korea. J Phys Therapy Sci.

[52] Park H, Oh H, Boo S (2019). The Role of Occupational Stress in the Association between Emotional Labor and Mental Health: A Moderated Mediation Model. Sustainability.

[53] Freire C, Ferradás MDM, Valle A, Núñez JC, Vallejo G (2016). Profiles of psychological well-being and coping strategies among university students. Front Psychol.

[54] Guinot J, Chiva R, Roca-Puig V: Interpersonal trust, stress and satisfaction at work: an empirical study. Personnel Review 2014.

[55] Watson R, Hoogbruin AL, Rumeu C, Beunza M, Barbarin B, Macdonald J, McCready T (2003). Differences and similarities in the perception of caring between Spanish and UK nurses. J Clin Nurs.

[56] Salimi S, Azimpour A, Mohammadzadeh S, Fesharaki M (2014). Psychometric properties of Persian version of the Caring Dimension Inventory (PCDI-25). Iran J Nurs Midwif Res.

[57] Tarbiyat NS, Salimi S, Feizi A: Predictors of nursing care behaviors in critical care units. 2019;17(5):371–8.

[58] Moattari M, Abedi H (2008). Nursing students’ experiences in reflective thinking: A qualitative study. Iran J Med Educ.

[59] Begat I, Ellefsen B, Severinsson E (2005). Nurses’ satisfaction with their work environment and the outcomes of clinical nursing supervision on nurses’ experiences of well-being–a Norwegian study. J Nurs Manag.

[60] Visser MR, Smets EM, Oort FJ, De Haes HC (2003). Stress, satisfaction and burnout among Dutch medical specialists. CMAJ.

